# Incidence and Risk Factors of Soil‐Transmitted Helminths Among Vegetable Farmers of Addis Ababa, Ethiopia

**DOI:** 10.1155/jotm/9138685

**Published:** 2026-08-12

**Authors:** Bethelhem kinfu Gurmassa, Sirak Robele Gari, Ephrem Tefera Solomon, Bitwe K. Dessie, Bezatu Mengistie Alemu

**Affiliations:** ^1^ Africa Centre of Excellence for Water Management (ACEWM), Addis Ababa University, Addis Ababa, Ethiopia, aau.edu.et; ^2^ CIMA/ARNET Centre for Marine and Environmental Research/Aquatic Research Network, Universidade do Algarve, Campus de Gambelas, Faro 8005-139, Portugal, ualg.pt; ^3^ College of Health and Medical Sciences, School of Medical Laboratory Sciences, Haramaya University, Harar, Ethiopia, haramaya.edu.et; ^4^ Water and Land Resource Centre of Addis Ababa University, Addis Ababa, Ethiopia; ^5^ Institute of Water, Environment and Climate Research, Addis Ababa University, Addis Ababa, Ethiopia, aau.edu.et

**Keywords:** exposed, nonexposed, STH, vegetable farmers, wastewater irrigation

## Abstract

**Background:**

Soil‐transmitted helminth (STH) infections are common neglected tropical diseases in areas with poor sanitation. In Ethiopia, urban farmers using contaminated river water for irrigation are exposed to parasite infection. This study examined the incidence and associated risk factors of STH infections among vegetable farmers in Addis Ababa, comparing those using wastewater with those using clean water.

**Methods:**

A mixed‐methods design was applied, incorporating a community‐based cross‐sectional survey and a longitudinal laboratory investigation from December 2021 to December 2023. The study involved 290 farmers (136 wastewater‐exposed and 129 unexposed) across 10 sites along the Big and Little Akaki Rivers. Data were collected via questionnaires, observation, health diaries, and stool samples, with diagnosis performed using the Kato–Katz method. STATA 14 was used for data analysis, including logistic regression to identify significant risk factors (*p* < 0.05).

**Results:**

STH incidence was significantly higher among wastewater‐irrigating farmers (19.8%) compared to nonusers (8.5%), with incidence rates of 7.1 and 3.04 per 1000 person‐months, respectively. The relative risk was 2.32, indicating wastewater irrigation increases STH infection. Risk factors among wastewater‐exposed farmers, poor fingernail hygiene (AOR = 2.25; 95% CI: 1.65–42.7), and not wearing boots (AOR = 3.4; 95% CI: 1.66–11.5) increased STH infection risk. Defecating on farms raised infection odds in both exposed (AOR = 2.89; 95% CI: 1.55–3.05) and unexposed (AOR = 1.92; 95% CI: 1.55–2.10) groups. Hand contamination (AOR = 6.9; 95% CI: 2.92–55.7), washing vegetables with wastewater (AOR = 11.6; 95% CI: 3.64–23.9), and not wearing protective clothing (exposed: AOR = 1.97; 95% CI: 1.11–87.9; unexposed: AOR = 1.89; 95% CI: 1.31–22.8) also significantly contributed to STH infection.

**Conclusion:**

Wastewater irrigation significantly elevates the risk of STH infections, promoting affordable safe wastewater irrigation practices (sand or gravel filters, drip irrigation) along with hygiene, sanitation, and health education for farmers, to protect farming communities.

## 1. Introduction

Soil‐transmitted helminth (STH) infections are among the most prevalent neglected tropical diseases (NTDs) [1]. It is affecting over 1.5 billion people globally, which represents about 24% of the world’s population, particularly, in low‐ and middle‐income countries [2]. These infections are predominantly found in tropical and subtropical regions where poverty, inadequate sanitation, and poor hygiene are common [3]. The main STH parasites that infect humans include *Ascaris lumbricoides, Trichuris trichiura*, and the hookworms *Necator americanus* and *Ancylostoma duodenale* [2]. STH parasite use wastewater to carry their fecal eggs onto vegetables and agricultural soil. Continuous wastewater irrigation keeps the soil’s moisture and organic content high, which creates favorable conditions for the survival of all STH species and turns irrigated fields into persistent sources of infection. *A. lumbricoides* and *T. trichiura* are primarily spread by ingestion of contaminated soil and uncooked or partially cooked vegetables, whereas hookworm larvae flourish in these environments and infect farmers by skin penetration during barefoot agricultural activities. Thus, wastewater irrigation transforms the farm into a focal point of infection, where the water, soil, and vegetables act as a continuous reservoir for both larval penetration and oral–fecal transmission. These parasites are a major public health concern, causing significant socioeconomic burdens [4]. In sub‐Saharan Africa, STHs represent a large share of NTDs, with Ethiopia being notably affected, having an estimated 36 million people infected [5].

Studies have illustrated that STH infections are a common health risk in areas where untreated wastewater is used for irrigation [6–9]. Wastewater irrigation is becoming common among urban farmers worldwide [10, 11]. The continuous supply of wastewater enables urban farmers to cultivate year‐round. Besides its perennial flow, the high nutrient content minimizes fertilization needs and consequently reduces input costs [12]. Although wastewater use offers numerous social and economic benefits, it also poses significant risks, particularly in terms of the prevalence of STH infections [13, 14]. High‐risk groups include farmers who frequently come into contact with wastewater, their families, nearby communities exposed to wastewater irrigation, and consumers of wastewater‐irrigated produce.

In Ethiopia’s capital city, Addis Ababa, the Little and Great Akaki Rivers were clean and used for cultivating various vegetables along the riverbanks for decades during the early days of the city’s establishment [15, 16]. However, in recent times, these rivers have become heavily polluted due to the discharge of industrial toilet waste and domestic sewage directly into the river [17–20]. Previous research has documented STH contamination in irrigation systems [13, 14, 21–23]. However, these investigations have primarily focused on measuring prevalence rates in the polluted river water while critical gaps remain. Although some studies have investigated exposure pathways, the direct impacts on farming communities and the specific infection risks among agricultural workers have not been adequately quantified. A comparative analysis of risk factors between wastewater and nonwastewater irrigation systems is essential to identify and mitigate contributors to STH transmission. Moreover, the odds of STH infection among farmers remain unknown. Understanding these risks is critical not only for agricultural workers but also for their families’ health. Investigating the risk factors of STH in both wastewater and nonwastewater irrigation farmers is essential to eradicate potential risk factors contributing to STH prevalence. This study aims to assess the incidence and risks of STH among wastewater and nonwastewater‐irrigating farming households in Addis Ababa.

## 2. Materials and Methods

### 2.1. Study Area

**FIGURE 1:**
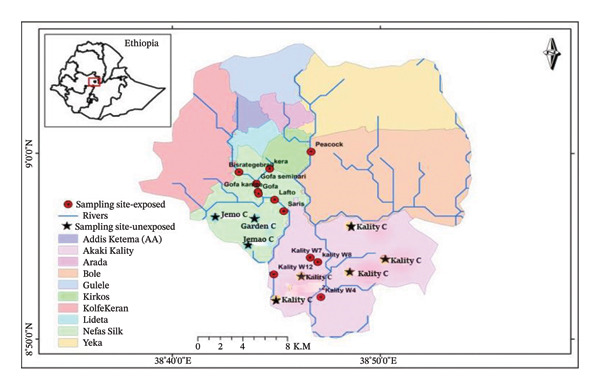
Map of the study area and the sampling sites (Jemo C and Kality C plots of farm land irrigated by nonwastewater). Source: Ali et al. [24].

This survey was conducted at 10 urban farming sites (wastewater and nonwastewater vegetable‐irrigating farming homes) along the two rivers, the Big Akaki and Little Akaki Rivers in the capital city, Addis Ababa, Ethiopia (Figure 1). According to the World Population Review, the population in 2024 is estimated at 5,703,628. Its geographic area has rapidly expanded over the last decade, from 31.05 km^2^ in 1974 to 365.18 km^2^ in 2020 [25].

The two rivers (Big and Little Akaki) are highly polluted, and water has been used to produce more than 1240 ha of leafy and root vegetables within and around Addis Ababa. Since the 1940s, this agricultural system has supported more than 1260 farming households in the city and at its periphery [26]. Almost all these farmers use polluted rivers, accounting for about 61% of the city’s vegetable supply and 90% of the leafy vegetable supply [27–29].

Urban agriculture has gained nationwide attention, with residents growing primarily fast‐growing leafy vegetables using various materials, from small pots to unused land or backyards. In this study, residents from Jemo and Kality Woreda eight and twelve were included as the unexposed group because the farmers there used piped and groundwater for producing leafy vegetables. The exposed group encompasses prominent vegetable production sites, known locally as Bisrategebrail, Gofa, Lafto, Saries, and Kera. They are all irrigated by the Little Akaki River; Peacock‐Urael and Akaki are irrigated by the Great Akaki River. These are studied under the exposed group (Figure 1). These sites are located in three subcities: Nifas Silk Lafto, Bole, and Akaki Kality.

### 2.2. Study Design and Period

Two study designs were used in the investigation. First, from September to December 2021, a community‐based cross‐sectional survey was undertaken to examine baseline conditions, such as water, sanitation, and hygiene (WASH) practices, farm‐related activities, and the baseline status of STH infections among study participants. Stool samples were collected and examined at baseline to determine the initial infection status and to identify individuals who were free from STH infection. Second, laboratory‐based longitudinal investigations were carried out from December 2021 to December 2023 among the same cohort of participants. Repeated stool examinations were carried out during the follow‐up period to monitor changes in infection status and to identify incident STH infections among individuals who were negative at baseline.

### 2.3. Population

#### 2.3.1. Source Population

The study’s source population consisted of all vegetable farming households along the Akaki River bank using wastewater irrigation, as well as vegetable farming communities in the subcities of Nifas Silk Lafto, and Akaki Kality in Addis Ababa that utilize alternative water source for irrigation.

### 2.4. Study Population

The study population included purposively sampled vegetable farmers from both wastewater and nonwastewater irrigation farmers. These farmers were selected based on their willingness to voluntarily provide consent to participate in the survey for a lengthy period of time.

### 2.5. Inclusion and Exclusion Criteria

The study inclusion criteria are as follows: vegetable farmers who use the Akaki River for irrigation are classified as the exposed group, while those using other water sources are classified as the unexposed group. Additionally, participants must have a subcity identification card and have lived in the area for more than 10 years. Individuals who are unwilling to provide stool samples and who have taken antihelminthic treatment within 28 days before data collection are excluded from the study.

### 2.6. Sample Size

The sample size was calculated using Epi‐Info Version 7 statistical software (Stat Calc) with a double proportion formula to determine participants between exposed and unexposed farming households. The final sample size was determined by considering the following factors: 80% power; a ratio of unexposed to exposed (1:1); a 95% confidence interval; an incidence of exposure among the exposed group of 18.5%; and an incidence of exposure among the unexposed group of 6.9% [30]. The final estimated sample size was 145 exposed and 145 unexposed participants. The final response rates were 93.7% (136/145) for the exposed group and 88.9% (129/145) for the unexposed group.

### 2.7. Sampling Technique

**FIGURE 2:**
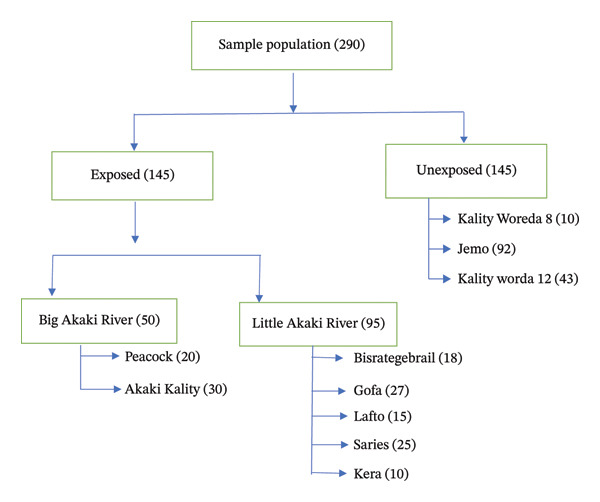
Proportional allocation of study population vegetable farmers in Addis Ababa, Ethiopia.

A multistage sampling technique was used to pick farmers from three subcities (Nifas Silk Lafto, Bole, and Akaki Kality). In the first step, seven of the districts in these subcities were purposefully chosen as wastewater irrigation vegetable farmers (exposed group) and three were chosen as nonwastewater irrigation vegetable farmers (unexposed group). In the second stage, the sample size was randomly assigned to the selected districts (Figure 2). Finally, 290 vegetable farmers representing 120 households participated in the study. The sample size was allocated to districts based on homes located near farms.

### 2.8. Study Variables

The study investigated STH infections as the dependent variables. The independent variables encompassed a range of factors, including sociodemographic characteristics, WASH conditions, and farming practices.

### 2.9. Data Collection

Data were collected using structured questionnaires and observation methods, focusing on predictor factors that could potentially expose the study participants to STH by trained data collectors. The data were collected from wastewater‐ and nonwastewater‐irrigating farming households (farmers). Wastewater‐irrigating farming households/farmers are exposed groups using highly contaminated Akaki Rivers for irrigation, whereas nonwastewater‐irrigating farming households/farmers are unexposed groups using nonwastewater sources of irrigation, including groundwater and pipe water, for growing vegetables. The questionnaire was first prepared in English and then translated into the local language (Amharic). It was then back‐translated to English by an anonymous translator to ensure the consistency of the two versions.

The longitudinal data were collected through health diaries, self‐reporting sheets, and stool samples from the study participants over a 3‐month period during each vegetable growing season, with the study spanning from December 2021 to December 2023. The primary aim of the health diary was to generate unbiased individual‐level information on the main disease outcome of interest. The self‐reporting sheets were used to monitor the participants’ exposure to STH occurrences.

### 2.10. Stool Sample Collection and Examination

Stool samples were examined using the Kato–Katz technique to determine STH infection. This method was selected due to its standard application in epidemiological studies and its ability to detect and quantify helminth eggs. The stool samples were collected using clean, leakproof, and screw‐cup containers and transported to the laboratory of EPHI using an icebox with frozen icepacks. The specimens were processed within 2 h of receipt or kept in an icebox where travel time exceeded 2 h.

The double Kato–Katz technique was performed as follows: A small amount of stool sample was pressed through a sieve to remove large particles. Part of the sieved stool was then transferred to the hole of a template on a slide using a flat‐sided spatula. The hole was filled; the template was removed; and the remaining sieved sample was covered with cellophane, which had been presoaked in glycerol. Then, the microscope slides were inverted, and the fecal samples were firmly pressed against the hydrophilic cellophane strip on another microscope slide or on a smooth, hard surface. The fecal material was spread evenly between the microscope slide and the cellophane strip; it should be possible to read newspaper print through the smear after clarification. The slide was carefully removed by gently sliding it sideways to avoid separating the cellophane strip or lifting it off. Then, the slide was placed on the bench with the cellophane upward and water‐evaporated while glycerol clears the smear, and finally, the smears were examined in a systematic manner and the number of eggs of each species reported [31].

### 2.11. Data Quality Assurance

Adequate training was given to data collectors and supervisors on techniques of interviewing and general approaches to the community. Before commencing the study, a pilot study was conducted on 5% of the calculated sample size at a nearby farming site that was not included in the study to test and validate the proposed questionnaire. Amendments were subsequently made to the data collection tool as per the finding of the pretest. The questionnaires were checked for missing data that were obtained the next day by data collectors. Supervisors also checked the accuracy of data by randomly interviewing some of the participating households.

The accuracy of the laboratory results was controlled by correctly labeling, calibrating equipment, and following standard operating procedures. The laboratory data collection sheet was used to collect lab data, which was then quickly entered into EpiData Version 3.1. Errors were corrected by revisiting the original questionnaire and responses. Microsoft Excel was used to construct the figure.

### 2.12. Data Analysis

The data were analyzed using STATA Version 14.0. Frequency tables were used to summarize the sociodemographic characteristics of the study participants, sanitation and behavioral characteristics, occupational exposure factors, and magnitude of STH. Bivariate analysis and multivariable analysis were used to estimate the crude odds ratio (COR) and adjusted odds ratio (AOR), respectively, in order to determine the relationship. Bivariate analysis was employed to identify the factors associated with STH at *p* < 0.05 without controlling confounders, whereas in the multivariable analysis, the association between exposure factors and STH was examined by controlling for potential confounders [32, 33].

The multicollinearity of variables was tested by calculating the variance inflation factor (VIF). From the AOR analysis, variables with a *p* < 0.05 were considered statistically significant and independently associated factors with STH. Morbidity due to STH infection among the study population was determined using a commonly employed measure of morbidity for cohort studies: disease incidence. Two types of incidences were calculated from the cohort study: incidence proportion and incidence rate, which were determined using the following common formulae.

(1) incidence proportion=number of new cases of disease during a specified period total population at risk at the start of the period×1000,

(2) incidence rate=number of new cases of disease during a specified period total person−time at risk during that period×1000.

Person‐time was obtained by summing the periods of observation for all individuals in the population at risk during the study period. In this study, the total number of people was the total number of individual subjects making up the cohort from each household, while the time was in month. Adding the person‐month of all rounds creates the total number of person‐month observed per household, thus allowing the calculation of the incidence rate over the entire reporting period and longitudinal comparison between month and study groups. This provided insight into the temporal variations of STH events between wastewater and nonwastewater irrigation farmers.

The likelihood that untreated wastewater farming household members who were exposed to a risk factor developed STH, as compared to nonwastewater irrigation farming households, was determined by using relative risk or risk ratio (RR), rate ratio, or absolute risk. To calculate the relative risk or rate ratio, a binary variable structure was produced. The relative risk was derived from the difference in the proportion of sick people between the wastewater‐exposed and nonexposed populations (nonwastewater irrigation farming households).

(3) relative risk= cumulative incidence for wastewater irrigation farming householdscumulative incidence for clean water irrigation farming households.

## 3. Results and Discussion

### 3.1. Sociodemographic Characteristics

In this study, 265 vegetable farming households were included (136 wastewater‐irrigating as exposed and 129 nonwastewater‐irrigating as unexposed farming households). The sociodemographic and economic characteristics of the study participants and their association with the odds of STH among farmers are given in Table 1. Nearly 69.8% of exposed farmers and 16.2% of unexposed farmers were male, with the majority aged 31 to 40. Female farmers in both categories had significant associations with the incidence of STH. Additionally, exposed participants above the age of 50 exhibited significant associations with the incidence of STH (COR: 1.80, 95% CI = 1.18, 2.01). In the unexposed category, 59.6% of participants went to elementary‐high school, whereas 38.2% of exposed participants did not attend formal education. Participants who did not attend formal education in both groups had a considerably higher incidence of STH (exposed [COR: 4.91, 95% CI = 1.55, 25.6] and unexposed [COR: 3.5, 95% CI = 1.11, 18.5], respectively).

**TABLE 1:** Bivariate analysis of sociodemographic and economic factors of Addis Ababa, Ethiopia (*n* = 265).

Factors	Category	Proportion (%)		COR (95% CI)	
		Exposed	Unexposed	Exposed	Unexposed
Age	< 30	5 (3.68)	42 (32.56)	1	1
	31–40	50 (36.76)	50 (38.76)	1.45 (1.29, 1.77)	0.25 (0.12, 0.45)
	41–50	42 (30.88)	32 (24.81)	1.68 (1.33, 1.92)	0.50 (0.20, 0.77)
	> 50	39 (28.68)	5 (3.88)	1.80 (1.18, 2.01)^∗^	0.76 (0.32, 0.98)

Sex	Male	95 (69.85)	21 (16.28)	1	1
	Female	41 (30.15)	108 (83.72)	1.86 (1.10, 2.12)^∗^	1.58 (1.42, 12.5)^∗^

Education	No formal education	52 (38.24)	15 (11.64)	4.91 (1.55, 25.6)^∗^	3.5 (1.11, 18.5)^∗^
	Elementary–high school	33 (24.26)	77 (59.69)	2.34 (1.55, 10.72)	1.5 (1.1, 7.21)
	Certificate and diploma	35 (25.74)	23 (17.82)	0.96 (0.10, 0.99)	0.10 (0.5, 0.29)
	Degree and above	16 (11.76)	14 (10.85)	1	1

Family size	< 3	23 (16.91)	79 (61.24)	1	1
	4–6	84 (61.76)	33 (25.58)	1.58 (1.22, 7.25)	0.38 (0.12, 0.89)
	> 6	29 (21.32)	17 (13.18)	2.76 (1.54, 15.9)	0.90 (0.32, 0.99)

Income (ETB)	< 1000	35 (25.74)	15 (11.63)	2.94 (1.55, 4.33)^∗^	1.89 (1.48, 34.7)
	1001–3000	44 (32.35)	37 (28.68)	1.35 (1.05, 27.9)	0.88 (0.11, 0.96)
	3001–5000	26 (19.12)	58 (44.96)	1.15 (0.7, 1.89)	0.45 (0.05, 0.82)
	> 5000	31 (22.79)	18 (14.73)	1	1

Marital status	Single	4 (2.94)	18 (13.95)	3.05 (1.22, 9.02)	4.15 (1.36, 25.5)
	Married	125 (91.91)	96 (74.42)	1	1
	Divorced	3 (2.21)	11 (8.53)	2.56 (1.02, 18.9)	0.76 (0.11, 0.84)
	Widowed	2 (2.94)	4 (3.10)	1.05 (1.02, 23.8)	0.48 (0.8, 0.85)

^∗^ *p* value < 0.05.

The incidence of STH infection in families with more than six members was 2.76 times greater in the exposed group (COR: 2.76, 95% CI = 1.54, 15.9) than in the unexposed group, which had an odds ratio of 0.90 (OR: 0.90, 95% CI = 0.32, 0.99). The majority of families in the exposed group with monthly incomes less than 1000 Ethiopian Birr (ETB) were infected with STH at a rate of 2.94 times (COR: 2.94, 95% CI = 1.55, 4.33), whereas the unexposed group had an odds ratio of 1.89 (COR: 1.89, 95% CI = 1.48, 34.7). The majority of participants in both groups were married, but the incidence of STH was higher among farmers who were divorced or single.

### 3.2. STH Infection Frequency Among Exposed and Unexposed Vegetable Farmers

#### 3.2.1. Incidence Proportion/Incidence Rate

**FIGURE 3:**
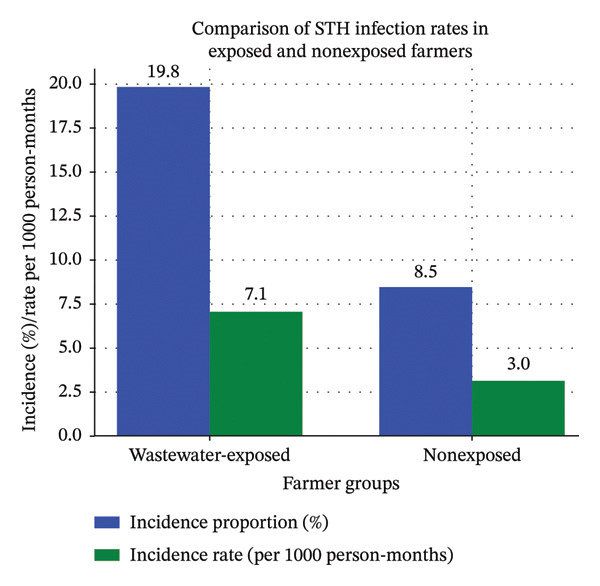
Comparison of helminth infection rates in exposed and nonexposed farmers.

In this study, the incidence proportion of STH infections was markedly higher among individuals exposed to wastewater, with 19.8% of subjects in this group reporting infections. More than 75% of the infected farmers were female and over 40 years of age. This figure was more than twice that observed in the unexposed farmer group, where only 8.50% of individuals reported STH infection (Figure 3).

The incidence rate, which is the number of new cases per population at risk in a given time period, also differed significantly between the two groups. The incidence rate among exposed farmers was 7.1 (Figure 3). In contrast, the unexposed group had a lower incidence rate of 3.04, showing the elevated risk associated with wastewater exposure. During investigation, the incidence rate of STH was calculated as 1000 people at risk each month. STH infection in the exposed participants occurred in all of the 3‐month recall periods for 3 years. Although the incidence of STH infection fluctuates considerably over time in both groups, the difference becomes more noticeable among nonwastewater users. The incidence rate among wastewater‐exposed farmers ranged from 0.0068 (6.89 per 1000 person‐months at risk) to a maximum of 0.0689 per person‐months at risk (68.9 per 1000 person‐months at risk) during vegetable planting and harvesting seasons.

The likelihood that untreated wastewater users exposed to a risk factor acquired STH as compared to nonwastewater‐irrigating farming families was calculated using relative risk. The relative risk of STH infection, calculated as the ratio of cumulative incidence of exposed to unexposed farmers, was 2.32, indicating that using wastewater for irrigation increases the risk of STH infection by 2.32 times compared to farmers utilizing nonwastewater sources for irrigation. The rate ratio was calculated using the incidence rate to determine the risk of STH infection caused by wastewater irrigation practice compared to nonexposed groups. Farmers using wastewater for irrigation had 2.36 times the rate of STH infection as compared to farmers using nonwastewater for irrigation, resulting in a risk difference of 0.0068 per 1000 person‐months excess of STH infections among the wastewater‐using farmers.

*WASH* factors for the incidence of STH among wastewater and nonwastewater‐irrigating farming communities:

The study identified several key factors influencing the incidence of STH among both exposed and unexposed irrigating farming communities. The majority of the exposed farming community (78.7%) lacked access to water near the toilet, whereas 18.6% of the unexposed community lacks access to water near the toilet. The odds of STH among wastewater users without access to toilet water were 1.75 times (COR: 1.75, 95% CI: 1.33, 8.98) higher than those with access (81.4%) who had a lower odds ratio of 1.28 (COR: 1.28, 95% CI: 1.10, 10.8) (Table 2). Similarly, the exposed farmers 9.56% used soap to wash their hands after using the toilet, but the unexposed farmers’ majority 37.21% used soap after using the toilet. The likelihood of STH infection among exposed farmers from Table 2 was 1.90 times higher (COR: 1.90, 95% CI: 1.11–18.9) than among the unexposed group, who had a 1.30 times increased chance of STH incidence (COR: 1.30, 95% CI: 1.23–19.7).

**TABLE 2:** Bivariate analysis of WASH determinant factors of STH (2021 Dec.–2023 Dec.).

Factors	Categories	Proportion (%)		COR (95% CI)	
		Exposed	Unexposed	Exposed	Unexposed
Water availability near the toilet	No	97 (78.68)	24 (18.60)	1.75 (1.33, 8.98)^∗^	1.28 (1.10, 10.8)^∗^
	Yes	29 (21.32)	105 (81.40)	1	1

Use of soap after toilet	No	123 (90.44)	81 (62.79)	1.90 (1.11, 18.9)^∗^	1.30 (1.23, 19.7)^∗^
	Yes	13 (9.56)	48 (37.21)	1	1

Share toilet with other households	No	15 (11.03)	90 (69.77)	1	1
	Yes	121 (88.97)	39 (30.23)	1.6 (1.2, 1.79)	1.08 (1.1, 4.82)

Defecate on the farm	No	107 (78.68)	125 (96.90)	1	1
	Yes	29 (21.32)	4 (3.10)	1.98 (1.52, 9.12)^∗^	1.53 (1.22, 71.5)^∗^

Bathing after farm work	No	115 (84.56)	80 (62.02)	1.82 (1.42, 2.63)^∗^	1.20 (1.55, 29.4)
	Yes	21 (15.44)	49 (37.98)	1	1

On‐site hand washing after work with soap	No	56 (41.18)	10 (7.75)	3.21 (1.41, 8.45)^∗^	1.36 (1.73, 21.8)
	Yes	80 (58.82)	119 (92.25)	1	1

Fingernails	No	74 (54.81)	105 (81.40)	4.08 (2.33, 91.02)^∗^	1.98 (1.81, 38.9)
	Yes	61 (45.19)	24 (18.60)	1	1

Always wash feet after work	No	56 (41.18)	33 (25.58)	1.75 (1.51, 8.23)	0.35 (0.14, 0.89)
	Yes	80 (58.82)	96 (74.42)	1	1

Wear boots/shoes at work	No	72 (52.94)	49 (37.98)	11.2 (2.39, 52.1)^∗^	2.1 (1.23, 9.56)
	Yes	64 (47.06)	80 (62.02)	1	1

^∗^ *p* value < 0.05.

The study also found that 88.97% of exposed farmers shared toilets with other households, higher than the 30.23% of unexposed farmers who did so. Sharing facilities increased the risk of STH infection, with an odds ratio of 1.6 (COR: 1.6, 95% CI: 1.2–1.79), suggesting that shared sanitation facilities may elevate exposure to contaminated environments. Furthermore, open defecation on farms was more common among exposed farmers (21.32%) than unexposed farmers (3.10%), nearly doubling their infection risk (COR: 1.98, 95% CI: 1.52–9.12), which highlights open defecation’s contribution to infection rates. A large proportion of exposed farmers (84.56%) did not bathe after farm work, whereas only 62.02% of the unexposed group neglected this practice. The odds of STH infection for those who skipped bathing were 1.82 times higher (COR: 1.82, 95% CI: 1.42–2.63) than for those who did bathe, underscoring the importance of hygiene after farm exposure (Table 2).

Handwashing practices at work also influenced infection rates; only 41.18% of exposed farmers had access to on‐site handwashing with soap, compared to 92.25% of unexposed farmers. Without this facility, exposed farmers faced a 3.21 times higher likelihood of STH infection (COR: 3.21, 95% CI: 1.41–8.45), underscoring the protective role of handwashing facilities. Personal hygiene practices, such as fingernail cleanliness, also had a notable impact; 54.81% of exposed farmers did not keep clean fingernails, compared to 18.60% of unexposed farmers. The odds of STH infection were 4.08 times higher (COR: 4.08, 95% CI: 1.33–91.02) for those with unclean fingernails, suggesting that simple hygiene practices are critical for reducing infection risks (Table 2).

Other hygiene practices, such as washing feet after work, were also relevant. Among exposed farmers, 41.18% did not wash their feet after work, versus 25.58% of unexposed farmers, leading to a 1.75 times higher infection risk (COR: 1.75, 95% CI: 1.51–8.23). Finally, footwear use played a major role in exposure to contaminated soil, with 52.94% of exposed farmers not wearing boots or shoes, compared to 37.98% of unexposed farmers. This practice led to an 11.2 times higher risk of infection (COR: 11.2, 95% CI: 2.39–52.1), highlighting the importance of wearing protective footwear to minimize soil exposure.

The absence of water near the toilet, not using soap for hand washing after using the toilet, and defecating on the farm have shown a significant effect on the incidence of STH in both wastewater‐exposed and unexposed households. However, the absence of bathing after farm work, on‐site hand washing after work with soap, fingernail cleaning, and the lack of wearing boots at work were significantly associated with STH incidence only in the exposed farming community.

#### 3.2.2. Farming Practices Factors

The findings showed factors associated with the incidence of STH infections among exposed and unexposed farming communities. The majority of exposed participants reported high rates of hand contamination with soil and irrigation wastewater (92.6%), significantly increasing the risk of STH by a factor of 20.4 (COR: 20.4, 95% CI: 2.99, 203.2). In comparison, among the unexposed group, 89.9% reported hand contamination with soil and non‐/wastewater, with the odds of acquiring an STH infection being 1.93 times lower (COR: 1.93, 95% CI: 1.25, 2.63) (Table 3).

**TABLE 3:** Bivariate analysis of farming practices determinant factors of STH (2021 Dec.–2023 Dec.).

Factors	Categories	Proportion (%)		COR (95% CI)	
		Exposed	Unexposed	Exposed	Unexposed
Hand contamination with soil and irrigation non‐/wastewater	No	10 (7.35)	13 (10.08)	1	1
	Yes	126 (92.65)	116 (89.92)	20.4 (2.99, 203.2)^∗^	1.93 (1.25, 2.63)

Cloth contamination with non/wastewater	No	33 (24.26)	20 (14.73)	1	1
	Yes	103 (75.74)	109 (84.50)	2.79 (1.22, 5.58)	1.56 (1.19, 2.08)

Touch face with a dirty hand	No	62 (45.59)	37 (28.68)	1	1
	Yes	74 (54.41)	92 (71.32)	10.2 (1.96, 65.7)^∗^	3.02 (2.09, 91.8)

Bring unwashed farm tools to the home	No	20 (14.71)	119 (92.25)	1	1
	Yes	116 (85.29)	10 (7.75)	15.4 (5.33, 44.9)^∗^	1.12 (1.08, 2.87)

Use working clothes at home	No	76 (55.88)	120 (93.02)	1	1
	Yes	60 (44.12)	9 (6.98)	23.5 (13.5, 108.7)^∗^	6.12 (2.56, 18.8)

Wash hands with the non‐/wastewater	No	37 (27.21)	40 (31.01)	1	1
	Yes	99 (72.79)	89 (68.99)	1.99 (1.17, 106.8)^∗^	1.59 (1.11, 4.84)

Wash vegetables with non‐/wastewater	No	59 (43.38)	13 (10.08)	1	1
	Yes	77 (56.62)	116 (89.92)	3.34 (1.99, 89.7)^∗^	1.62 (1.22, 1.89)

Eat meals in the farm	No	19 (13.97)	117 (90.70)	1	1
	Yes	117 (86.03)	12 (9.30)	1.4 (1.1, 2.09)	0.45 (0.16, 0.69)

Eat food falling ground the in farm	No	119 (87.50)	125 (96.90)	1	1
	Yes	17 (12.50)	4 (3.10)	32.5 (5.36, 125.9)^∗^	7.26 (2.08, 145.1)^∗^

Walking on the farm with barefoot for irrigation	No	34 (25.0)	115 (89.15)	1	1
	Yes	102 (75.0)	14 (10.85)	8.6 (1.88, 10.5)^∗^	4.05 (1.99, 77.6)

Wearing protectives	No	130 (95.59)	35 (27.13)	2.99 (1.08, 6.98)^∗^	1.98 (1.64, 17.9)^∗^
	Yes	6 (4.41)	94 (72.87)	1	1

Time spent in farm in day	Half a day	9 (6.62)	59 (45.74)	2.78 (2.15, 4.05)	1.43 (1.13, 1.89)
	All the day	121 (88.97)	57 (44.19)	1.98 (1.33, 5.39)	1.50 (1.10, 1.77)
	4 h	6 (4.41)	13 (10.08)	1	1

^∗^ *p* value < 0.05.

Clothing contamination with wastewater also played a significant role, affecting 75.74% of the exposed group and 84.50% of the unexposed group. This exposure was associated with a 2.79 times higher risk of STH among the exposed group (COR: 2.79, 95% CI: 1.22–5.58). The analysis suggests that although contamination rates are higher in the unexposed group, the incidence rate is higher in the exposed group. This indicates that farming environments using wastewater irrigation are highly contaminated with STH. Similarly, touching face with dirty hands was a common behavior, associated with an increased risk of STH infection by 10.2 times higher for the exposed group (COR: 10.2, 95% CI: 1.96–65.7), compared to 3.02 times for the unexposed group, highlighting that face‐touching habits increase contamination risks, especially in wastewater‐exposed settings (Table 3).

Bringing unwashed agricultural tools home was a significant issue, with 85.29% of exposed farmers doing so compared to 7.75% of unexposed farmers. This behavior increased the STH risk for the exposed group by 15.4 times (COR: 15.4, 95% CI: 5.33–44.9), suggesting that cleaning tools before bringing them home could be an important preventative measure. Additionally, wearing work clothing at home was associated with a significant 23.5 times increase in STH risk (COR: 23.5, 95% CI: 13.5–108.7) for exposed farmers, who were more likely to be exposed to STH‐contaminated soil and wastewater. Moreover, the use of wastewater for handwashing and vegetable washing also posed risks. While 72.79% of the exposed group washed hands with wastewater, increasing their infection risk nearly twofold (COR: 1.99, 95% CI: 1.17–106.8), 56.62% of exposed farmers used wastewater to wash vegetables, which was linked to a 3.34 times increased infection risk (COR: 3.34, 95% CI: 1.99–89.7). These findings underline the necessity of clean water access for handwashing and food preparation, especially in wastewater‐exposed community (Table 3).

Other behaviors, such as eating meals or consuming food that fell on the ground while on the farm, were more common in the exposed group and associated with a significantly higher STH infection risk 32.5 times higher for those eating food off the ground (COR: 32.5, 95% CI: 5.36–125.9). Walking barefoot on the farm was a notable factor, with a 75.0% prevalence in the exposed group, increasing infection risk by 8.6 times (COR: 8.6, 95% CI: 1.88–10.5). Not wearing protective gear and spending the entire day on the farm also heightened STH risk, emphasizing the protective role of footwear and time management in reducing prolonged exposure.

From the identified risk factors in Table 3, eating food that falls on the farm and not wearing protective clothing increased the incidence of STH infection among both exposed and unexposed farming communities (*p* < 0.05). Cloth contamination with non‐/wastewater, eating meals on the farm, and time spent on the farm during the day were the only risk factors that did not have a significant effect on the incidence of STH among the exposed participants, while others listed in Table 3 had a significant effect.

#### 3.2.3. Multivariable Analysis of Nonwastewater‐ and Wastewater‐Related Determinants of STH

In the final multivariable regression, three models (M1: sociodemographic [AOR]; M2: water, sanitation, and hygiene [AOR]; and M3: farming practices factors [AOR]) were tested to identify the independent predisposing factors for the incidence of STH infection that remained significant after controlling for potential confounders. Each model examined the independent association between these categories of incidence factors and STH infection, adjusting for potential confounders. By considering the AOR of each of the three factors that are statistically significant, we then run the analysis for Model M4. In the fourth model, table four risk factors were independently associated with the incidence of STH infections. *From WASH practices* behavior, poor fingernail cleanness (AOR = 2.25; 95% CI = 1.65–4.27) and not wearing boots/shoes at work (AOR = 3.4; 95% CI = 1.66–11.5) (Table 4) increased the odds of STH incidence only among the wastewater‐exposed farmers. Defecating on the farm, increased the incidence of the infection among both exposed (AOR = 2.89; 95% CI = 1.55, 3.05) and unexposed (AOR = 1.92; 95% CI = 1.55, 2.10) (Table 4) farming communities. *From farming-associated factors*, hand contamination with soil and non‐/wastewater irrigation (AOR = 6.9; 95% CI = 2.92, 55.7), washing hands with the non‐/wastewater (AOR = 1.98; 95% CI = 1.45, 2.15), washing vegetables with non‐/wastewater (AOR = 11.6; 95% CI = 3.64, 23.9), and walking on the farm with barefoot for irrigation (AOR = 3.97; 95% CI = 1.54, 7.08) (Table 4) were significantly associated with the incidence of STH among the exposed farming community. From Table 4, not wearing protective clothing was identified as a significant risk factor for STH incidence in both the exposed community (AOR = 1.97; 95% CI = 1.11, 87.9) and the unexposed community (AOR = 1.89; 95% CI = 1.31, 22.8).

**TABLE 4:** Multivariable analysis of nonwastewater‐ and wastewater‐related determinants for the incidence of STH.

Factors	Categories	Model I, AOR ∗ (95% CI)		Model II, AOR ∗ (95% CI)		Model III, AOR ∗ (95% CI)		Model IV, AOR ∗ (95% CI)	
		Exposed	Un exposed	Exposed	Unexposed	Exposed	Unexposed	Exposed	Unexposed
Age	< 30	1						1	1
	> 50	1.86 (1.28, 2.11)^∗∗^						1.25 (1.05, 1.87)	1.1 (1.09, 2.06)

Sex	Male	1	1					1	1
	Female	1.96 (1.19, 2.42)^∗∗^	1.78 (1.49, 129.2)^∗∗^					1.86 (1.12, 1.89)	1.57 (1.10, 1.87)

Education	No formal education	8.01 (1.45, 35.2)^∗∗^	5.5 (1.15, 38.5)^∗∗^					2.5 (1.42, 3.43)	1.79 (1.29, 2.05)
	Degree and above	1	1					1	1

Income (ETB)	< 1000	3.44 (1.35, 4.63)^∗∗^						1.90 (1.11, 2.06)	1.5 (1.1, 2.21)
	> 5000	1						1	1

Defecate on the farm	No			1	1			1	1
	Yes			1.99 (1.42, 9.22)^∗∗^	1.69 (1.27, 71.8)^∗∗^			2.89 (1.55, 3.05)^∗∗^	1.92 (1.55, 2.10)^∗∗^

On‐site hand washing after work with soap	No			4.27 (1.21, 8.95)^∗∗^				1.94 (1.11, 11.9)	1.6 (1.1, 6.21)
	Yes			1				1	1

Fingernails	No			2.78 (1.73, 9.02)^∗∗^				2.25 (1.65.4.27)^∗∗^	1.9 (1.2, 12.8)
	Yes			1				1	1

Hand contamination with soil and irrigation non‐/wastewater	No					1		1	1
	Yes					15.7 (3.89, 313.2)^∗∗^		6.9 (2.92, 55.7)^∗∗^	2.5 (1.9, 8.6)

Use working clothes at home	No					1		1	1
	Yes					13.5 (3.95, 118.7)^∗∗^		8.14 (3.99, 94.2)	1.45 (1.2, 3.12)

Wash hands with the non‐/wastewater	Yes					5.99 (1.77, 156.8)^∗∗^		1.98 (1.45, 2.15)^∗∗^	1.35 (1.09, 1.92)
	No					1		1	1

Wash vegetables with non‐/wastewater	Yes					9.33 (2.99, 97.8)^∗∗^		11.6 (3.64, 23.9)^∗∗^	2 (1.9, 4.21)
	No					1		1	1

Walking on the farm with barefoot for irrigation	Yes					18.6 (2.78, 203.5)^∗∗^		3.97 (1.54, 7.08)^∗∗^	1.51 (1.06, 3.32)
	No					1		1	1

Wearing protectives	Yes					1	1	1	1
	No					3.97 (1.88, 77.8)^∗∗^	2.78 (1.44, 20.9)^∗∗^	1.97 (1.11, 87.9)^∗∗^	1.89 (1.31, 22.8)^∗∗^

Abbreviation: AOR, adjusted odds ratio.

^∗∗^ *p* < 0.05.

## 4. Discussion

A prospective cohort study was used to assess the incidence of STH disease and associated determinant factors among vegetable farmers utilizing wastewater and nonwastewater irrigation methods. The findings revealed that the incidence of STH among farmers using wastewater for irrigation was 19.8%, which is significantly higher compared to nonwastewater sources 8.50%. The higher probability of infection among wastewater users was associated with the presence of helminth contamination of irrigation wastewater and the parasite persistence in the environment with their ability to cause infection even at minimal levels of exposure [34].

The incidence rate of STH infections among farmers who used wastewater for irrigation ranged from 6.89 to 68.9 per 1000 person‐months at risk, which is notably higher than that of nonwastewater‐irrigating farmers, ranging from 6.89 to 20.7 per 1000 person‐months at risk. Research has shown that untreated or inadequately treated wastewater can harbor a high concentration of helminth eggs, leading to significant health risks for those who come into contact with it during agricultural activities [5, 14]. For instance, a study indicated that farmers utilizing wastewater for irrigation had a prevalence ratio of 1.50 for hookworm infections compared to those who did not use wastewater, highlighting the direct correlation between wastewater exposure and STH infection rates [23, 35]. Moreover, the method of irrigation plays a crucial role in the level of contamination; practices that allow for direct contact with wastewater, such as the furrow or flood irrigation used by farmers in this study, further amplify the risk of STH transmission among farmers [7, 13].

Moreover, the study has identified potential risk factors for STH infection. These included defecating on the farm, hand contamination with soil and irrigation wastewater, inadequate cleansing of fingernails, hand and vegetables wash with the wastewater, walking on the farm with barefoot, and failure to wear protective clothing or footwear. After controlling for all STH predictors, the socioeconomic and demographic factors between the exposed and unexposed farmers were not significant. The differences in STH infection between exposed and unexposed farmers could be because of the extra exposure to wastewater pathogens among the wastewater‐irrigating farmers. Consider two farmers from similar socioeconomic backgrounds: one who frequently works in areas irrigated with wastewater and the other who utilizes nonwastewater sources. Despite their similar social and economic conditions, the wastewater‐using farmer faces additional health risks through daily contact with contaminated irrigation water while performing routine tasks such as planting, weeding, and harvesting.

From WASH practices behavior, the study identified a concern environmental issue that affects both groups: the widespread habit of open defecation near farming areas. This approach introduces a common risk factor that affects all farmers, regardless of irrigation practices. In this case, when community members practice open defecation on or near the agricultural land, the soil becomes contaminated with helminth eggs and larvae. During rains or agricultural activity, these parasites spread across the farming area, creating multiple transmission routes for helminths infections [36–38]. The condition is especially problematic in the study areas where sufficient sanitation facilities are few or far from the farming area. The farm workers lack access to toilet facilities during their long working hours in the fields, and they have no option but to practice open defecation. A study conducted in Kumasi, Ghana, found that farmers and their family members exposed to wastewater irrigation were three times more likely to be infected with *A. lumbricoides* and hookworm compared to a control group of nonfarmers, highlighting the significant contribution of wastewater irrigation to STH transmission [38].

Occupational exposures such as hand contamination with soil and irrigation wastewater, inadequate cleansing of fingernails, and washing hands and vegetables with the wastewater expose the farming community to odds of helminth infection. Consider a typical workday scenario: Farmers often handle soil with their bare hands while planting, weeding, or harvesting, and their fingernails frequently harbor contaminated soil particles due to insufficient cleaning practices. Additionally, many farmers, due to limited access to clean water sources, are forced to use the same wastewater for multiple purposes from irrigating their vegetables to washing harvested vegetables and even cleaning their working tools during fieldwork. This creates a risky cycle of repeated exposure to parasitic organisms [23].

The Food and Agriculture Organization stated in a 2019 study that certain occupational hazards offer particularly serious risks during specialized farming operations. When farmers harvest root vegetables such as carrots or potatoes, they frequently come into close touch with contaminants in the soil, and washing these vegetables in wastewater adds to the exposure risk [39]. Research findings have consistently demonstrated increasing levels of helminth eggs present in vegetables grown using wastewater irrigation systems [5, 13, 21].

This finding suggests that the odds of helminth infections are significantly higher among farmworkers who do not wear protective gear compared to those who take precautions by wearing appropriate protective gear. Specifically, the use of gloves has been shown to reduce the risk of infection from helminth eggs by acting as a barrier that prevents these parasites from penetrating the skin or attaching to the nails of farmers [40]. This is particularly important, as helminth eggs can easily be ingested through hand‐to‐mouth contact during routine agricultural activities, such as eating or touching the face after handling contaminated soil or vegetables. By wearing gloves, farmworkers can minimize direct contact with contaminated surfaces, thereby reducing the likelihood of transferring infectious agents to their mouths. This protective measure is crucial in environments where exposure to contaminated irrigation water and soil is prevalent, as it helps to interrupt the transmission cycle of helminths [5, 41, 42].

The contrast becomes particularly evident when comparing the infection rates: Wastewater‐irrigating farmers without protective instruments show significantly higher infection rates compared to nonwastewater‐irrigating farmers without protective. Both groups of farmers benefit from glove usage. The protective effect is more important in wastewater irrigation settings where exposure risks are inherently higher. In nonwastewater‐irrigated farming settings, while the overall pathogen load is lower, the importance of protective equipment remains significant, albeit with different risk levels. When nonwastewater irrigation farmers wear gloves, they primarily protect themselves from soil‐dwelling parasites and any pathogenic micro‐organisms that might exist from environmental factors like open defecation near farming areas.

The strengths of the study were the inclusion of home visits to evaluate the household and socioeconomic conditions of farmer participants in relation to their helminth infection status. By conducting direct observations of sanitation infrastructure, hygiene practices, and living conditions at the farmers’ homes, we obtained a more comprehensive assessment of infection incidence compared to relying solely on survey data. The robust sample size of our study was adequately powered to estimate differences in STH infection rates between the exposed and unexposed farmer groups, allowing us to identify key determinants of STH infections with a high degree of statistical confidence. By leveraging both quantitative survey data and qualitative observations from home visits, strengthening the evidence base for targeted interventions aimed at reducing the burden of parasitic diseases in agricultural communities.

However, several limitations should be acknowledged. First, the retrospective nature of the study introduces the potential for recall bias, which may affect the accuracy of the data collected. Second, the study was conducted in a specific geographical area, which may limit the representativeness of the findings across all farming communities using wastewater for irrigation, as factors such as cultural practices, environmental conditions, and access to healthcare services can vary significantly between regions. These limitations may impact the generalizability of our conclusions, emphasizing the need for further research in diverse settings to validate our findings.

## 5. Conclusion

The findings of this prospective cohort study highlight the significant public health risks associated with the use of wastewater for vegetable irrigation, revealing a notably higher incidence of STH infections among farmers utilizing wastewater compared to those using nonwastewater sources. This elevated incidence is primarily attributed to the presence of helminth eggs in untreated and treated wastewater, which can persist in the environment and pose serious health risks to those who come into contact with it during agricultural activities.

Additionally, the study identified key risk factors for the incidence of STH infections, including open defecation practices near farms, occupational exposures such as hand contamination with soil and irrigation wastewater, washing hands and vegetables with wastewater, and inadequate hygiene practices such as fingernail cleanness, walking on the farm with barefoot, and not wearing protective clothing and footwear.

In conclusion, the findings of this study highlight the urgent necessity for improved wastewater management practices and focused interventions to diminish the burden of STH infections among farming communities that rely on wastewater irrigation. Implementing a multifaceted approach and encompassing enhanced awareness creation regarding personal hygiene, lifestyle, and environmental sanitation, as well as the use of protective equipment, can significantly contribute to disrupting transmission cycles, eliminating parasitic infections, and reducing the risk for these vulnerable populations. Furthermore, sustainably strengthening health extension programs and deworming programs within the communities is a pivotal strategy to eliminate STH and create a safer environment for farming activities.

## Author Contributions

Bethelhem Kinfu Gurmassa conceived and designed the study, carried out data collection and laboratory examination, and finalized the writing of the manuscript. Bezatu Mengistie Alemu, Sirak Robele Gari, Ephrem Tefera Solomon, and Bitwe K. Dessie designed the study and revised the manuscript. The drafted manuscript was revised.

## Funding

No funding was received for this manuscript.

## Disclosure

All authors read and approved the final manuscript.

## Ethics Statement

The Addis Ababa University College of Natural and Computational Sciences Institutional Review Board (IRB) Committee approved the research under Approval No. IRB/03/14/2022. Official permission letter was received from each municipal agriculture and health subcity admiration. After the objective and contents of the study were clarified to all participants, written consent was obtained from each participant. Confidentiality of the study results was maintained, and no other tests were done on the stool samples collected other than those mentioned in the data collection section.

Participants testing positive for STH at baseline were referred to local government health posts for treatment, as budget limitations precluded direct medication provision at that stage. During the follow‐up phase, however, the study provided medication directly to infected participants. These individuals were also advised to visit nearby government hospitals to ensure proper treatment and monitoring.

## Consent

Please see the Ethics Statement.

## Conflicts of Interest

The authors declare no conflicts of interest.

## Data Availability

The data that support the findings of this study are available on request from the corresponding author. The data are not publicly available due to privacy or ethical restrictions.

## Appendix A

**Operational definitions**

Farmers: those communities that cultivate vegetables.

Wastewater: the untreated Little and Grate Akaki River.

Nonwastewater: other water sources without wastewater.

Permanent: farmers who have lived along the Akaki Riverside for more than 10 years.

**Abbreviations**

STH: Soil‐transmitted helminths.

WHO: World Health Organization.

EPHI: Ethiopian Public Health Institute.

FDRE: Federal Democratic Republic of Ethiopia.

UNDP: United Nations Development Programme.
